# Can a Low-Budget Questionnaire Support Improved Health Service Accessibility and Sustainability: Results from an Exploratory Study

**DOI:** 10.1177/00469580261468784

**Published:** 2026-07-14

**Authors:** Stefan Hellstrand, Leif Sundberg, Jon Karlsson, Roy Tranberg, Ulla Hellstrand Tang

**Affiliations:** 1 Nolby Ekostrategi, Sweden; 2 Gothenburg Diabetes Association, Sweden; 3 The Department of Orthopaedics, Institute of Clinical Sciences, Sahlgrenska Academy, 3570 University of Gothenburg, Gothenburg, Sweden; 4 The Department of Prosthetics & Orthotics, The Department for Biomedical Engineering and Medical Physics, Sahlgrenska University Hospital, Gothenburg, Sweden

**Keywords:** sustainable development, health service accessibility, healthcare, travel time, diabetes, diabetic foot, non-communicable diseases, sustainable development goals

## Abstract

**Introduction:**

In sustainable development, time is a physically limited resource affecting economic and social sustainability. In healthcare systems, the time of healthcare staff is linked to economic constraints, while the time patients spend travelling and receiving treatment affects social sustainability, including access to healthcare services. Reducing time costs in healthcare may contribute to several of the 17 Sustainable Development Goals (SDGs) within Agenda 2030. This exploratory study aimed to: (i) probe whether a low-budget questionnaire combined with standardised data processing could estimate time costs for patients, their employers, and healthcare personnel associated with a healthcare visit; (ii) generate initial estimates of sustainability impacts of time costs, in physical and monetary terms.

**Methods:**

The study explored whether this low-budget approach could generate results of sufficient quality to inform choices aimed at improving healthcare accessibility. Patients with diabetes attending a healthcare facility for therapeutic footwear completed a questionnaire regarding time spent travelling and treatment. Healthcare staff time was recorded. Results were analysed and presented in physical and monetary terms.

**Results:**

The questionnaire generated information that was processed into estimates of parameters of strategic importance for choices supporting sustainability and access in healthcare systems in urban and rural areas. For the 101 patients included, the total time used was 360.6 hours, of which 23% represented employers’ time, 49% patients’ private time, and 28% healthcare staff time. The total estimated monetary value of time consumed was SEK 142,911 (USD 13,466), with 33% related to employers’ time, 13% to patients’ time, and 54% to healthcare staff time. The estimated mean cost per visit ranged from SEK 1,233 (USD 116.2) to SEK 1,944 (USD 183.2).

**Conclusions:**

The low-cost questionnaire and data-processing approach appears promising for estimating time costs for patients, employers, and healthcare staff, thereby supporting decisions aimed at improving healthcare accessibility and sustainability.

## Highlights

• Time-related barriers to healthcare remain insufficiently examined. Time spent by patients on travel, waiting, and treatment, as well as time lost by employers and time used by healthcare personnel, generates costs that are rarely quantified or considered in healthcare planning. This limits the ability to assess efficiency, equity, and sustainability in healthcare systems and hinders progress towards the Sustainable Development Goals (SDGs).
• This study examines whether a low-cost survey approach can generate data of sufficient quality to capture time-related costs from a multi-actor perspective. Focusing on patients with diabetes receiving therapeutic footwear, the study quantifies time use for patients, employers, and healthcare staff in both physical and monetary terms.
• The study demonstrates how time-related costs for patients, employers, and healthcare providers can be integrated into decision-support frameworks. This information may support the identification of measures to improve healthcare accessibility, cost-efficiency, equity, and sustainability.

## 1. Introduction

### 1.1. Healthcare, Diabetes, and Sustainability

The availability of healthcare depends, at least partially, on the time it takes for the patient to travel to healthcare providers. Limited access to healthcare has a negative effect on a sustainable development, as expressed by United Nations (UN) Sustainable Development Goal 3: to ensure healthy lives and promote well-being for all people of all ages.^1^ Measuring sustainability performance in healthcare is crucial for resource allocation and efficient use of resources and human efforts.^2^ That generates information by which healthcare providers can adapt to changing environmental, economic, and social conditions, securing continued service delivery, not least for persons living with chronic diseases such as diabetes.^3,4^

To evaluate how travel time affects the sustainability of healthcare including accessibility, trustworthy data on travels to and from healthcare providers are needed. In the present study, we probe whether answers to a questionnaire filled in by patients visiting a Department of Prosthetics and Orthotics (DPO) were sufficient to collect data on travels to and from a DPO. An advantage is that the costs for collecting and processing data can be kept low, while the results obtained can help increasing accessibility to healthcare, given that the estimates have a sufficient quality. This relates to an element in participatory rural appraisal and agroecology called optimal ignorance. By optimal ignorance trade-offs can be optimised, where costs for gathering and processing data are hold down, while the usefulness of results are sufficiently good to support decision-making.^5,6^ The same approach is found in Impredicative Loop Analysis (ILA)^7^: ILA is a methodological contribution within post-modern science, for analysis of performance in complex systems. Health of humans belong to this class, so does agroecosystems. In the analysis of a specific agricultural subsystem in its mutual biophysical and socioeconomic contexts, information of different subsystems at different system levels, and their dependencies are needed. When searching the needed data of constants and relations, following a dictum attributed to Keynes, it is better to be approximately right than precisely wrong. In ILA one element to secure that results are approximately right is to in an iterative way probe already known results against data from reality.

The questionnaire covered data on the type of transportation chosen, distances, fuel use, and time used. In the present study, we focus on the time aspect.

Diabetic foot ulcers (DFUs) are common among patients with diabetes and often leads to severe complications, negatively affecting patients’ quality of life and leading to substantial socioeconomic costs.^8^ To minimise these costs, access to healthcare that helps prevent DFUs is essential.^3^

The meaning of ‘sustainable development’ differs between contexts. Hellstrand presented a toolbox for sustainable development by an integration of concepts from economy, systems ecology, and agricultural sciences^9^ and Hellstrand et al^2^ have adopted this toolbox for the evaluation of sustainability performance in healthcare systems.

### 1.2. Sustainability and Healthcare

The general toolbox referred to above has been used in studies of patients with diabetes, who run the risk of developing DFUs.^2^ The authors concluded that by using the tools in this toolbox, the impact of choices in healthcare on the 16 national environmental quality objectives in Sweden,^10^ as well as a number of the UN’s 17 Sustainable Development Goals (SDG),^1^ can be evaluated.

There are three production factors in economic theory; labour, capital, and land. Land is a synonym for nature, or, in modern terminology, the ecological dimension of the economy. Gross domestic product (GDP) is the value of the production of goods and services, labour relates to the instrumental value of humans in the GDP economy, i.e., how humans contribute to value in GDP terms. This is done in two ways, both through the quantity of their time that they sell to their employers, and through the quality of the same time. Assuming that everything else is kept constant, the amount of labour, i.e., the time the labour force is available in the economic system, sets the limits for production, which in turn limits the welfare level that can be sustained, including the volume of healthcare that can be funded. An improvement in GDP results in greater resources being available at the level of the national economy, and some of those gains could in turn be made available for public welfare systems.

It is worth mentioning that (while not contributing to GDP) the amount of labour, as defined above, is not just constrained by the monetised labour contributing to GDP but could also involve substitution from non-marketed labour such as unpaid activities like household work, childcare, caring for relatives, volunteering.

If there is a way of performing a certain task in healthcare that uses less of the personnel’s time, more patients can be treated within the same budget restriction. If, by the same measure, the time that the collective of patients must take off from their employers decreases, their employers’ contribution to GDP increases. As the funding of public welfare systems, including healthcare, is limited by the size of the economy, an improvement in GDP results in improved funding for public welfare systems.

For society, the production factor ‘labour’ is a function of all the available time of the individuals in the society. Within healthcare systems, the time of the employees is one of multiple factors limiting the capacity to fulfil the needs of patients.^11^ Moreover, the time it takes for patients to access healthcare is critical for its availability.^12^ Nature sets two types of restrictions on the society; the time, and the capacity of nature to provide natural resources.

In Impredicative Loop Analysis (ILA),^7^ the physical limitedness of (i) ecological resources, as well as the limitedness of (ii) time, is integrated into multi-scale, multi-criteria assessments of the sustainability performance of systems. Time in ILA is a parameter that links system performance at individual level to national and global levels. It can be considered as having a mosaic effect that links system performances between micro and macro levels, and further between subsystems.^2^

In health economics two approaches commonly used to value time are the Human Capital Approach (HCA)^13^ and the Friction Cost Method (FCM).^14^ In HCA, the estimate of costs is based on estimated gross earnings, including payroll taxes and other employer-paid benefits, i.e., the full cost of employee compensation. Sometimes only reported hourly earnings (wages and salaries), excluding benefits, are used to estimate productivity losses. In FCM it is the costs before a vacancy is filled by a previously unemployed person that is measured.

### 1.3. Travel Time and the Availability of Healthcare

Travel time to healthcare facilities may constrain human well-being,^15^ as it affects accessibility to healthcare.^16,17^ Persons living in non-urban areas are more likely to have a lower access to healthcare as a function of distances in the geography and in time, compared with persons living in urban areas.^17,18^ Functional limitations may also cause decreased access to healthcare due to increased travel time compared with patients where such limitations are absent.^19^

In the current study, an analysis – based on real-world data from standard treatments at DPOs – was performed of the time consumed by patients, their employers, and the healthcare providers, respectively, in connection with the provision of healthcare to patients with diabetes who are in the need of early, preventive healthcare.

### 1.4. Aims

The study had two aims.

(i) To evaluate the capacity of a low-budget questionnaire to collect useful data on the time cost of patients, their employers, and healthcare staff, for patients visiting a healthcare provider.
(ii) To utilise the collected data in a first evaluation of the sustainability impacts of the time used, in physical (hours) and monetary (SEK and USD) terms.

This exploratory study was guided by the assumption that a reduction of the time used by the patients, the healthcare personnel, and the employers of the patients, is essential when improving sustainability in healthcare, everything else equal.

### 1.5. Perspectives and Theoretic Background

Within different disciplines, contributions have been made that provide tools and concepts supporting a sustainable development. Some examples are from economic theory, systems ecology, applied environmental science, post-normal science, and agricultural sciences. As mentioned above, Hellstrand^9^ and Hellstrand et al^2^ integrated element from these disciplines into a toolbox, supporting the evaluation of the sustainability performance of different systems/activities in the three sustainability dimensions – the ecological, economic, and social dimensions. The tools were adopted for different tasks, still in its basic structure internally coherent.

Within economic theory, the sustainability concept has roots going back to Hicks,^20^ who can be said to be the founding father of the economic models used in resource accounting. He discussed income as net return on the total capital stock. Weitzman^21^ developed this theoretical framework, showing how net domestic product (NDP) under certain conditions can be interpreted as a perpetual (sustainable) income. Hartwick^22^ showed that a sustainable income stream required that all returns from expropriating non-renewable resources were reinvested in (preferably renewable) capital, for the total capital stock not to decrease.^20^ In Hartwick’s^23^ and Mäler’s^24^ studies, the accounting rules for the use of renewable and non-renewable resources were established.

In the field of ecological economics that emerged around 1990, there was a concern that common economic models at that time focused on the production factors ‘labour’ and ‘capital’, while mainly ignoring nature, i.e., the production factor ‘land’, as it was called in classical economics. Scientists trained in economy as well as in systems ecology, started to cooperate to gain deeper insights into the conditions for a sustainable development. Some of these early efforts discussed the ambition in terms of once again placing the production factor ‘land’ in focus in economic theory and practice. Examples of these models are the studies by Daly and Cobb^25^ and Daly.^26^

Hernández-Blanco and Costanza^27^ provided a description of the development of economic theory – from classic economic theory, which treated the production factors ‘land’, ‘labour’, and ‘human-made capital’, to neoclassic economics, which focused on labour (human capital) and man-made capital such as buildings, physical infrastructure, and machines, and finally to the new field of ecological economics emerging around 1990. In ecological economics these three production factors once again become important, the production factor ‘land’ being, in this context, synonymous with the ecological dimension of the economy. In ecological economics, natural capital, human capital, and manufactured or built capital, were important, and a fourth capital form, social capital, was soon added. OECD^28^ developed its own version of what sustainable development is. They stressed the three dimensions of sustainable development, i.e., the ecological, economic, and social dimensions, and the need to keep track of the four types of capital: natural, man-made, human, and social capital.

Giampietro^7^ developed a methodology within post-normal sciences, called ILA. In this approach, time is a fundamental dimension when evaluating the sustainability of systems. Hernández-Blanco and Costanza^27^ also stress time as an essential dimension to handle when evaluating the sustainability performance of systems.

## 2. Materials and Methods

This exploratory study was guided by the assumption that a reduction of the time used by patients, the healthcare personnel, and the employers of the patients, everything else equal, is essential when improving sustainability in healthcare.

By following the steps described below, the two aims outlined were met, and first results were generated. If this exercise to a low cost provided estimates in a structure that was relevant for an evaluation of the impact of the time factor in healthcare, and if the quality of the estimates generated was sufficient, then this study is one step further in the development of analytical and management tools that can contribute to an increased access to healthcare for people in urban and rural areas. If so, it is a contribution to increased sustainability in healthcare.

### 2.1. Questionnaire

Patients with diabetes visiting one of four different DPOs in a region in the western part of Sweden, participated in a survey approved by the Gothenburg Regional Ethical Board (Reg. No. 1041-13).

The study was carried out following the Code of Ethics of the World Medical Association (Declaration of Helsinki) for experiments involving humans.^29^ The participants received written and oral information about the study, including information relating to their right to withdraw from the study at any time without explanation. Participants were included after they signed a written consent form. The four DPOs participating in the study were located in Borås, Göteborg, Skövde, and Trollhättan, and the number of participating patients in Borås was 15, in Göteborg 40, in Skövde 24, and in Trollhättan 22, amounting to a total of 101 patients. All the DPOs are in Region Västra Götaland.

The survey included, first of all, a test of foot status.^30,31^ The second part was a questionnaire. It covered the following three areas; the perceived quality of the visit and the treatment, the way the patient travelled to and from the DPO, and the time that the visit consumed for the patients and their employers.

The questionnaire was generated by a multi-professional team including representatives from Göteborgs Diabetesförening (the Gothenburg Diabetes Association), a regional organisation for patients with diabetes. Appendix 1 presents the questions related to the time aspect.

### 2.2. Patients

Patients eligible for inclusion visited a DPO for the provision of therapeutic footwear. The criteria for inclusion were patients 18 years or older, diagnosed with diabetes, and understanding Swedish.

**Figure 1.**
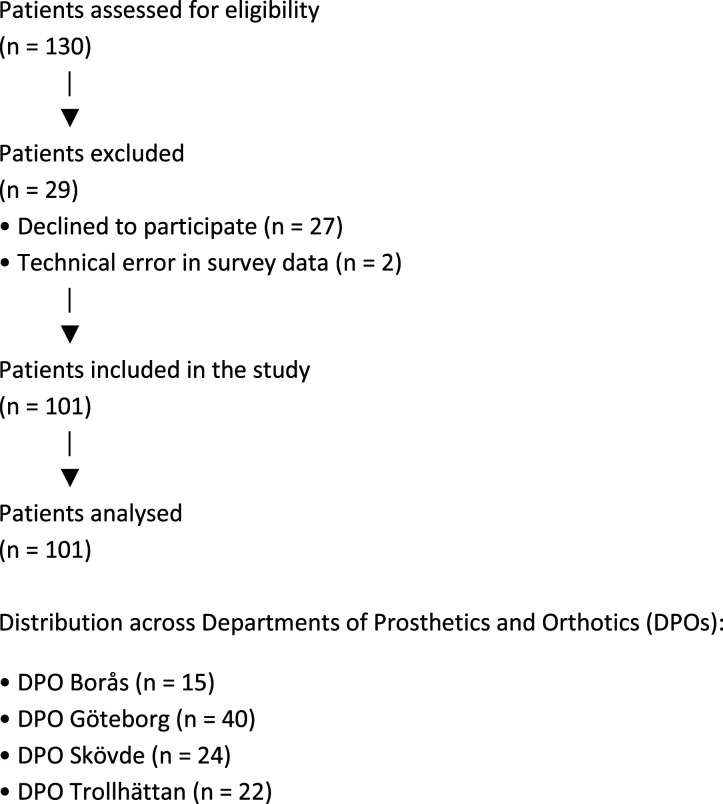
Participant flow through the study. of 130 patients assessed for eligibility, 29 were excluded (27 declined participation and 2 were excluded due to technical errors in the survey), leaving 101 patients who were included and analysed. Participants were recruited from four departments of prosthetics and orthotics (DPOs).

The number of patients asked to participate was 130, of which 27 declined to participate, and 2 was excluded due to technical errors, leaving 101 participants in the study (Figure 1). The rationale for the exclusion was to reduce bias and maintain the integrity of the statistical analyses.^32^ Of 101 participants, 34 had diabetes type 1 and 67 had diabetes type 2, and 45 participants were women and 56 were men. The definition of gender (female/male) was generated automatically based on the personal number which is unique to each patient and provided for all Swedish people by the Swedish Tax Agency. The mean duration ± standard deviation (SD) of the disease was 17 ± 14 years.

We used the STROBE reporting guideline to draft this manuscript, and the STROBE reporting checklist when editing, included in supplement A.^33^

### 2.3. Monetary Value of Time

Time-related data were collected partly as continuous values (e.g., absence from work reported in hours and minutes) and partly as categorical intervals (e.g., travel time). For travel time, reported intervals were converted into continuous values using the midpoint of each interval. For example, responses such as “1–2 hours” were assigned a value of 1.5 hours. For open-ended categories (e.g., “more than 4 hours”), a conservative midpoint assumption was applied. Total travel time was defined as the round-trip time to and from the DPO, as specified in the questionnaire. The total time consumption per visit included travel time, time spent at the DPO (treatment), and waiting time, as reported by the patients.

The results of the evaluation of the monetary cost of time are presented at the 2018 price level. The cost of time to patients is estimated by multiplying the hours consumed by the price per hour. This price is a weighted average for travelling by private car from ASEK 7.0 (abbreviation of “Analysmetod och samhällsekonomiska kalkylvärden” (Analysis method and socioeconomic calculation values)).^34^ ASEK is used in Sweden in analyses of the welfare-economic impact of investments in the physical transport infrastructure, including environmental impacts, health impacts and impacts on time consumed in different alternatives. The cost per hour of the staff at the DPO in 2018 was 760 SEK. The time consumed by the patient’s employer is valued as the average contribution to GDP in Sweden in 2018, as the total GDP^35^ divided by the total number of labour hours.^36^

### 2.4. Statistics

Mean values for each of the four DPO units are presented, as well as the overall mean value of all the treated patients. As this study is explanatory, it is not suitable for advanced statistical analysis. When estimating the average time consumed per visit by patients, employers, and healthcare staff at four Departments of Prosthetics and Orthotics, 95% confidence intervals were calculated for time consumed by patients and employers of the patients, as this was data that was collected by the questionnaire. The value for time consumed by the healthcare staff was based on an assumption of the authors, therefore a confidence interval for this parameter is irrelevant.

The size of the study population was not based on a power calculation because the study was a part of a larger study, an intra-rater reliability study, originally being dimensioned to assess the intra-reliability regarding the risk of developing DFU.^30,31^

## 3. Results

Of the total time consumed, 81.4 hours accounted for the employers’ time (23%), 178.2 hours for the patients’ time (49%), and 101.0 hours for the time spent by the personnel at the DPO (28%) (Table 1).

**Table 1.** Total time Consumed by Patients, Employers, and Halthcare Personnel at Four Departments of Prosthetics and Orthotics in Region Västra Götaland

​	Time consumed, hours				
	n	Employers’ time	Private time	At DPO	In total
DPO Borås	15	7.5	26.0	15.0	48.5
DPO Göteborg	40	22.6	71.0	40.0	133.6
DPO Skövde	24	14.3	38.7	24.0	77.0
DPO Trollhättan	22	37.0	42.5	22.0	101.5
Total	101	81.4	178.2	101.0	360.6

Information of the average time per visit for the mentioned categories is presented in table 2. The results are provided for each DPO and as averages over the four DPOs.

**Table 2.** Average Time Consumed per Visit by Patients, Employers, and Healthcare Personnel at Four Departments of Prosthetics and Orthotics in Region Västra Götaland

​	*Time consumed, hours*			
	Employers’ time	Private time	At DPO	In total
DPO Borås	0.5	1.7	1.0	3.2
DPO Göteborg	0.6	1.8	1.0	3.3
DPO Skövde	0.6	1.6	1.0	3.2
DPO Trollhättan	1.7	1.9	1.0	4.6
Total	0.8	1.8	1.0	3.6
95% confidence interval	0.36	0.23	Not applicable	0.28
*Results in %*				
​	22.6	49.4	28.0	​

*Note.* In the column ‘At DPO’ one hour was set for each visit and therefore no confidence interval was calculated.

The estimated total monetary value of time consumption was 142,911 SEK (13,466 US$) (Table 3). Of this total, 33% relates to employers, 13% to patients, and 54% to the DPOs (Table 4). Thus, close to half of the total value (33% + 13% = 46%) is attributable to patients and their employers.

**Table 3.** Monetary Value of Time Consumption for Visits to Four Departments of Prosthetics and Orthotics (DPOs) in Region Västra Götaland

​	*Costs SEK 2018*				*Costs USD 2018*			
	Employers’ time	Private time	At DPO	In total	Employers’ time	Private time	At DPO	In total
DPO Borås	4 383	2 709	11 400	18 492	413	255	1 074	1 742
DPO Göteborg	13 207	7 397	30 400	51 004	1 244	697	2 864	4 806
DPO Skövde	8 376	4 028	18 240	30 645	789	380	1 719	2 888
DPO Trollhättan	21 623	4 428	16 720	42 770	2 037	417	1 575	4 030
Sum	47 590	18 561	76 760	142 911	4 484	1 749	7 233	13 466

**Table 4.** Average Monetary Cost of Time Consumption per Visit at Four Departments of Prosthetics and Orthotics (DPOs) in Region Västra Götaland

​	*Costs SEK 2018*				*Costs USD 2018*			
	Employer’s time	Private time	At DPO	In total	Employer’s time	Private time	At DPO	In total
DPO Borås	292	181	760	1 233	27.5	17.0	71.6	116.2
DPO Göteborg	330	185	760	1 275	31.1	17.4	71.6	120.1
DPO Skövde	349	168	760	1 277	32.9	15.8	71.6	120.3
DPO Trollhättan	983	201	760	1 944	92.6	19.0	71.6	183.2
Sum	471	184	760	1 415	44.4	17.3	71.6	133.3
Total, in per cent	33.3	13.0	53.7	​	33.3	13.0	53.7	​

**Figure 2.**
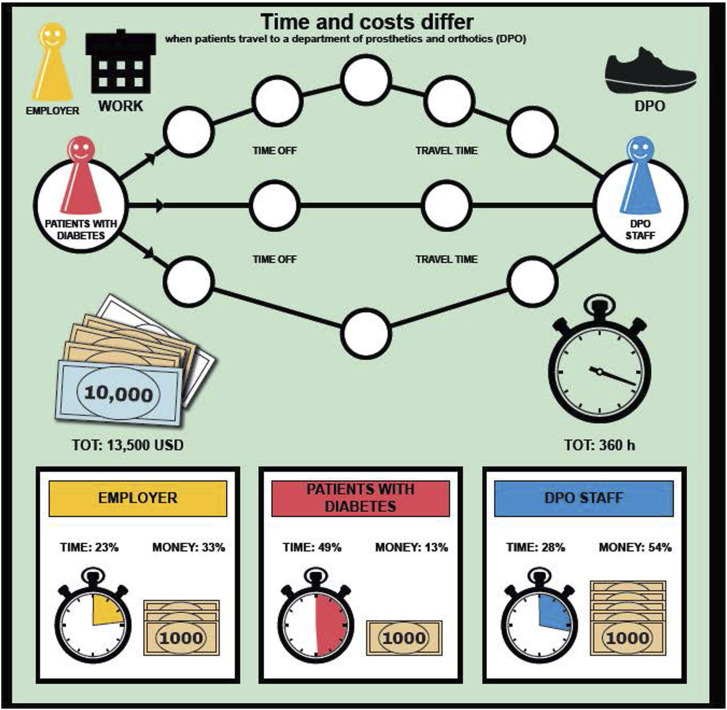
Distribution of time consumption and associated costs for visits to departments of prosthetics and orthotics

The results presented in Tables 1-4 are illustrated in Figure 2, entitled *Distribution of time consumption and associated costs for visits to Departments of Prosthetics and Orthotics*.

It shall be noted that staff time and employer cost estimates are partly assumption-based, i.e. partly not a function of the data gathered by the questionnaire.

## 4. Discussion

### 4.1. Material and Method

Data are from 2014. Can this be relevant in 2026? To answer this, we return to the aims of the study.

(i) To evaluate the capacity of a low-budget survey to gather useful data on the time cost of patients, their employers, and healthcare staff, when patients visit a healthcare provider.
(ii) To utilise the collected data in a first evaluation of the sustainability impacts of the time used, in physical (hours) and monetary (SEK and USD) terms.

We conclude that the data from 2014 still are of relevant as the transportation patterns as of 2014 are still dominating. The organisation of healthcare related to prosthetics and orthotics in the studied region has remained largely unchanged since 2014.

The questionnaire collected discrete data via choices of intervals. In the analysis this was transformed to continuous data, assuming that every data point in an interval corresponded to the medium of the interval. The reason for this choice was to simplify the filling in of answers.

The reason why the study assumed a fixed value for the personnel per visit was that this was the time allocated per visit for the staff. To hold the costs for the study low, we assumed that this was a good estimate with no variation for all visits.

Compared with the HCA and the FCM we used another way to estimate the marginal loss of productivity when patients were absent from work. In HCA, the productivity loss is estimated as the loss of gross earnings over the lifetime of the individual. In FCM the estimate relates to the cost of finding someone that can replace the absent worker.

The HCA-estimate is only a partial measure of the productivity loss for the employer, and for society. GDP measures the sum of costs for personnel, costs for depreciation, and the result of companies. For public sector, the contribution to GDP is assumed to equal the total costs of the public sector. The measure being used in the present study, the contribution to GDP per hour worked in the economy captures the productivity loss to the economy due to the impact on the production both via a lower input of labour, and that the man-made capital is underutilised in proportion to the lower input of labour.

### 4.2. Was the Questionnaire Sufficiently Good?

In this study, information about the time consumed by a visit to a DPO, was collected by a quite simple questionnaire. Information about time costs is essential in an evaluation of the accessibility and the sustainability of healthcare. Efficient methods for obtaining accurate information about the time factor are crucial. Here, “efficiency” is understood somewhat imprecisely as the fraction between the value of the information obtained and the costs incurred in obtaining it. Although the questionnaire used in this study was simple, it nevertheless provided useful information. Its simplicity may also be regarded as a quality in itself, contributing to lower costs and making it more user-friendly. As to whether the questionnaire yielded valuable information, the answer is yes: it proved sufficient for the purposes of the study. At low cost, it generated data of importance for further analysis.

### 4.3. Costs and Benefits for Different Actors

The time consumed by patients, their employers, and the healthcare provider, for a visit to healthcare, should be considered when improving the sustainability of healthcare. By using a limited set of questions, valuable information has been collected. Through the collection of real-world data, time costs for different groups can be estimated and compared.

It should, furthermore, be mentioned that the same questionnaire gives information about the travels to and from the healthcare provider. This information makes it possible – by following the routes used by the Swedish Environmental Protection Agency in their official publications regarding emissions of greenhouse gases and emissions to air – to cover 20 emissions contributing to climate change, ozone close to the ground, eutrophication, acidification, direct health impacts, and emissions of nine different metals, with their own, individual unique spectrum of environmental and human health impacts. Energy use for transportations was also recorded in this section of the questionnaire. Moreover, the questionnaire included a section in which the patient indicated how they perceived the quality of the visit and the treatment received.

Thus, the costs of performing the data collection were shared across three different areas in which the collected information proved valuable data, with time use being one of them.

The time costs of visits to healthcare are valuable information in the design of healthcare at different system levels. One case in point is a new way of organising the provision of insoles to patients with diabetes, described by Hellstrand et al, and where the time per treatment was close to halved, as were the associated ecological, economic, and social costs.^2^ Without increasing the costs of the healthcare provider, the number of patients receiving the early, preventive treatment increased from 5%, to almost 10%. This will have positive impacts in the future through a decrease in DFU and amputations, thereby decreasing future costs in the healthcare system, improving the quality of life of patients with diabetes, and improving the productivity for the collective of patients with diabetes. The new way of organising this care is now implemented.

### 4.4. Accessibility of Healthcare as a Function of Distance

At a higher system level, the information generated about the time factor supports decisions regarding the localisation of healthcare, taking into account ecological, economic, and social sustainability aspects.^1,3^

Von Thünen in 1826 made an important contribution regarding the rational localisation of production in a rural economy.^37^ In the same work he made an early and substantial contribution to human capital theory.^38^ The limited access to time affected the rationale localisation of production, while the quality of the available time for work also affected the economic output, that is the quality of human capital. These relations between time and rational localisation of production in a rural society, and between the human capital (the quality of labour-time available) and production value was of interest in 1826. With current geopolitical and ecological challenges the same issues are of no less interest 200 years later, in 2026.

This study presents one way to collect and process real-world data about the time costs of an activity related to healthcare. Generated estimates provide information supporting the design of sustainable and accessible healthcare systems. Such information is important when deciding how to organise the everyday work, e.g., at a DPO. It is also valuable when deciding how to locate healthcare facilities in the landscape, supporting accessible, equal, and sustainable healthcare solutions for all.^39^

### 4.5. Is the Value of Human Time Relevant?

Measured in physical terms (hours), the patients’ private time represented 49% of the total time registered for visits. Measured in monetary terms, it represented only 13% of the value of the time consumed. This suggests that there is a need to explicitly show time in its physical dimension and as a monetary value, since this constitutes a basis in the development of accessible healthcare. According to Sui, the physical dimension of time stresses the importance of the perspective of time geography.^40^ It points to time as a mosaic effect that can bridge analyses in different subsystems as well as between scales.

### 4.6. Time Accounting Supports Measures that Increase Accessibility and Efficiency

The time cost for patients, and, when accurate, for their employers, was captured in physical (hours) and monetary (SEK and USD) terms (Tables 1-4, Figure 2). The results show that the time consumed by patients and their employers is substantial, measured in both physical and monetary terms. As demonstrated in previous work, this supports the identification of solutions that save time for patients, their employers, and the staff at the healthcare facility.^2^

The example given by Hellstrand et al,^2^ and referred to above, showed the value of time accounting. Measures increasing the percentage, from 5% to 9.3%, of patients that gained access to the necessary preventive treatment, within the same budget restriction, were identified. Such an improvement in accessibility will have an impact on the health status of the 5% of the population with diabetes in the region in question. It will (i) reduce future costs for the healthcare provider due to decreasing levels of DFUs and amputations, (ii) increase the productivity in healthcare in terms of the level of health of the population attained for the same amount of money, (iii) increase the productivity of the labour force in the regional economy when their general health status improves, and, not least, (iv) improve the life quality of these patients. To be clear, this is not a result of this study, but another, published study. It stresses the general importance of time-accounting in healthcare, where this study presents a low budget alternative for this.

This study stresses the time perspective in relation to the accessibility of healthcare. Analysing and visualising the time consequences of different options in healthcare, from the design of treatments at a low system level to the localisation of facilities for healthcare in the geographical setting, makes it easier to identify solutions with advantages like those mentioned above.

### 4.7. Limitations

One limitation of the study is the self-reported estimation of the time used for the visit. As shown previously, self-reported data are of great value, but their accuracy may vary.^41^ Moreover, the authors were unable to obtain the underlying calculation that formed the basis of the estimated value of an hour at the DPO (760 SEK, at the 2018 price level). A second limitation is the assumption that all the patients that would otherwise have been at work were employees, whereas, in reality, some might well have been running their own enterprises. A third limitation is that the analyses have been carried out without making sub-analyses of the time consumed by women and men, respectively. The reason for this is that the studied population only included 101 participants and that the number of participants visiting any of the four sites in 2014, when the data were collected, was a minimum of 15 and a maximum of 40. Further splitting these four groups into women and men would have yielded too small samples for further analysis. However, it would be interesting in further research to evaluate whether differences between women and men exist in terms of the time they spend visiting a healthcare provider and, if differences exist, the reasons for those differences. A further limitation is that no formal sample size or power calculation was performed, as the study was exploratory and based on data from a larger study. This may limit the statistical power and generalisability of the findings. Finally, the results are dependent on the assumptions applied in the estimation procedures. Future studies could explore the sensitivity of the results to alternative assumptions and valuation approaches. This relates mainly to the monetary valuation approach in this study.

### 4.8. Major Contribution

The major contribution of this paper is that it presents a method for gathering information about time costs in healthcare and processing the collected data into estimates that are valuable for decision-making aimed at improving sustainability and access to care for patients in both urban and rural areas. The costs are low, while the estimates, to our knowledge, provide information on strategically important parameters that seldom influence planning processes within healthcare systems, ranging from the organisation of everyday work at a DPO to the future localisation of hospitals and healthcare units within the wider geographical landscape.

## 5. Conclusions

The questionnaire in combination with data on time used by the health personnel staff was sufficiently good in combination with the path of processing the obtained data to estimates on the time costs of patients, their employers, and the healthcare provider for visits to healthcare, here four DPOs.

The same study design can be used for other forms of healthcare as well.

Real-world data are a limited asset in the design of sustainable, accessible, and equal healthcare to all.

## Supplemental Material

Supplemental Material - Can a Low-Budget Questionnaire Support Improved Health Service Accessibility and Sustainability: Results from an Exploratory Study Supplemental Material for Can a Low-Budget Questionnaire Support Improved Health Service Accessibility and Sustainability: Results from an Exploratory Study by Stefan Hellstrand, Leif Sundberg, Jon Karlsson, Roy Tranberg and Ulla Hellstrand Tang in Inquiry: The Journal of Health Care Organization, Provision, and Financing.

## Data Availability

The raw data supporting the conclusions of this article will be made available by the authors on request.*

## Appendix

### Appendix 1. A Survey of the Time Spent on the Visit

Patients answered the following questions related to the time associated with their visit to a DPO. They used a tablet to answer the questions.

1. Have you taken time off work for your visit to the Department of Prosthetics & Orthotics today?
  a. Answer: yes/no
2. If yes, how long do you estimate that you will be absent from work today?
  a. Answer: register hours and minutes
3. How long (in total, to and from) will transport to the Department of Prosthetics & Orthotics take today? Answer:
  a. Less than 1 hour
  b. 1-2 h
  c. 2-3 h
  d. 3-4 h
  e. More than 4 h

### Abbreviations

CPO: Certified prosthetist and orthotist
DFU: Diabetic foot ulcer
DPO: Department of Prosthetics and Orthotics
GDP: Gross domestic product
ILA: Impredicative Loop Analysis
MEA: Millennium Ecosystems Assessment
NDP: Net domestic product
OECD: Organisation for Economic Co-operation and Development
SEK: Swedish krona
USD: USA dollar.
